# Drugs inducing T-cell mediated cutaneous adverse reactions and contact sensitizers evoke similar responses in THP-1 cells

**DOI:** 10.1186/2045-7022-4-S3-P50

**Published:** 2014-07-18

**Authors:** Margarida Gonçalo, João Martins, Ana Silva, Bruno Neves, Teresa Cruz, Celeste Lopes

**Affiliations:** 1 Clinic of Dermatology, University Hospital and Faculty of Medicine of Coimbra, Portugal; 2 Faculty of Pharmacy, University of Coimbra, Center for Neuroscience and Cell Biology, Portugal; 3 University of Coimbra, Center for Neuroscience and Cell Biology, Portugal; 4 Center for Neuroscience and Cell Biology and University of Aveiro, Department of Chemistry, Portugal

## Background

Contact sensitizers induce an innate immune response in dendritic cells (DC) enhancing antigen presentation and T cell response. Little is known concerning the effect of systemic drugs causing T-cell mediated cutaneous adverse drug reactions (CARDs). Therefore, we studied the in vitro effect of drugs on THP-1 cells, a monocyte cell line widely used to show activation by contact sensitizers.

## Methods

THP-1 cells were stimulated for 24h with allopurinol (ALP), oxypurinol (OXP), ampicillin (AMP), amoxicillin (AMX), carbamazepine, sodium valproate (VAP), lipopolysaccharide (LPS), a DC maturation stimulus, and the strong contact sensitizer, 1-fluoro-2,4-dinitrobenzene (DNFB), at concentrations that reduced cell viability to 70%, evaluated by the Alamar Blue test. We studied p38 MAPK activation by Western Blot and the expression of DC maturation markers, pro-inflammatory cytokine/chemokines and heme oxygenase 1 (HMOX-1) genes by real-time RT-PCR.

## Results

All drugs significantly upregulated HMOX-1 gene (mean log2 values varying from 1.842 ± 0.164, p<0.01 for AMP to 3.096 ± 0.575, p<0.05 for OXP). All drugs, but the anti-epileptics, increased the pro-inflammatory chemokine IL-8/CXCL8 (mean log2 values varying from 0.959 ± 0.154, p<0.05 for AMX and 4.729 ± 0.508, p<0.05 for OXP). Both DC maturations makers (CD83 and CD40) were significantly upregulated by VAP and AMP (respectively 1.594±0.672, p<0.05 and 0.999±0.226, p<0.05) and CD83 was also upregulated by VAP. Other genes studied were irregularly activated. Moreover, like DNFB, all drugs activated p38 MAPK. In general, allopurinol and oxypurinol showed the most intense effect, very similar to DNFB and inferior to LPS. The concomitant stimulation of THP-1 cells by OXP and AMP had no additive effect on the endpoints studied.

## Discussion

Like contact sensitizers, systemic drugs activate THP-1 cells in vitro. Direct activation of monocytic or DC that participate in antigen presentation may be an important step in the pathophysiology of delayed immune mediated CADRs. Drugs use different signalling pathways and affect these cells with a different intensity that may reflect the frequency and severity of the CADRs they cause. The effect of systemic drugs on THP-1 cells needs to be further studied in order to confirm the usefulness of this in vitro test to study their sensitizing potential, similarly to its use for contact sensitizers.

